# Haemoglobinopathy Awareness among Young Students in Turkey: Outcomes of a City-Wide Survey

**DOI:** 10.1371/journal.pone.0159816

**Published:** 2016-07-22

**Authors:** Ramazan Azim Okyay, Özlem Çelenk, Ersin Nazlıcan, Muhsin Akbaba

**Affiliations:** 1 Department of Communicable Diseases, Ceyhan Community Health Center, Adana, Turkey; 2 Departmant of Projects, The Governorship of Hatay, Hatay, Turkey; 3 Department of Public Health, Çukurova University Faculty of Medicine, Adana, Turkey; University College London, UNITED KINGDOM

## Abstract

The success of prevention programs demonstrated the importance of raising awareness about haemoglobinopathies since the lack of knowledge and awareness about the disorders may serve as barriers to prevention, disclosure of disease status as well as to testing for haemoglobinopathies. The aim of this study is to investigate the knowledge and attitudes of middle and high school students towards haemoglobinopathies in Hatay, where the disorders are prevalent. This cross-sectional study was conducted on 8^th^ and 9^th^ grade students across Hatay including all sub provinces. From May 2012 to December 2012, a total of 1925 students filled the questionnaires which query the knowledge level and attitudes of students by face to face method. Among questions regarding students’ knowledge about haemoglobinopathies, the lowest correct response rate was observed in “How do these diseases transmit?” with 31.8%, meaning most of the students did not know that the diseases are transmitted by heredity. Significant differences were observed between the correct answer rates of the students and their status of being previously informed. Students who had a diseased person around were having a 2.597-fold (95%CI = 1.886–3.575); students possessing at least one parent at secondary education level or above were having a 1.954-fold higher probability of being previously informed (95%CI = 1.564–2.443). Due to the lack of knowledge about haemoglobinopathies in middle and high school students, we suggest health education programs including informative lectures particularly about the genetic basis of the disorders especially in the regions where the disorders are prevalent.

## Introduction

Haemoglobinopathies–that is to say, the thalassemia and sickle-cell disorder- are both recessive inherited and widely extended diseases [1]. There are not only nearly 240 million people throughout the world that are heterozygous for β-thalassemia but approximately 200,000 affected homozygotes are also born yearly [2]. Although nearly all different types of thalassemia can be seen in the whole world, it is more widespread in the regions of the Equator, Africa, Asia and the Mediterranean, in which Turkey is located as well [3].

Based on the survey conducted by the Ministry of Health and Turkish National Haemoglobinopathy Council in 2006, the estimated number of the carriers of thalassemia was 1,400,000 and the number of patients was about 5,000 in Turkey. Similarly, the overall frequency of β-thalassemia trait was calculated as 4.3% of 377,339 healthy subjects and Antalya (13.1%) and Cukurova regions (10%) had the highest prevalence of β-thalassemia trait according to the same survey [4].

In relation to other haemoglobinopathies, even though the carrier prevalence of sickle cell anemia is generally 0.37–0.60% in Turkey, a considerably higher frequency has been seen in regions such as the Cukurova region of the country (3.0–44.0%) [5].

Haemoglobinopathies do not have a definite medical care, thus the World Health Organization (WHO) has recommended a control program including enlightenment of the public, screening for carriers, antenatal detection and genetic counseling leading to the avoiding of birth of an affected child [6]. Henceforward premarital screening programs have been introduced worldwide in many of the affected countries. Several countries including Italy, Greece, Canada, the UK and Cyprus have benefitted from such programs. For example, Cyprus reduced the prevalence of thalassemia from extremely high to almost negligible levels following the introduction of a screening program [7].

The success of prevention programs in these countries has also demonstrated the importance of raising awareness about the disorders since the lack of knowledge and awareness about the disorders, their consequences, and psychosocial and cultural issues may serve as barriers to prevention, disclosure of disease status as well as to testing for haemoglobinopathies [2, 3].

In this paper, we investigated the knowledge and attitudes of middle and high school students towards haemoglobinopathies in Hatay, where the disorders are prevalent, with aims to determine their preventive behaviors, to measure the level of awareness about haemoglobinopathies and to give a lead to planning future educational interventions.

## Materials and Methods

### Study Design

This cross-sectional study aiming to investigate the knowledge and attitudes of middle and high school students towards haemoglobinopathies, was held with the participation of Directorate of Health, Eastern Mediterranean Development Agency and Thalassemia Association in Hatay which is a city in Cukurova Region. The study was planned to be conducted on 8^th^ and 9^th^ grade students across Hatay including all sub provinces.

At the time of the study there were 52055 students attending 8^th^ and 9^th^ grades in Hatay, which constituted the study universe. According to Ministry of Health data, the prevalence of abnormal hemoglobin carriers in Hatay is 15.1% in total [8]. Taking this rate into account, the sample size was calculated as 1986 students in 95% confidence interval and with 2% margin of error.

### Data collection

In the study, a questionnaire which queries the knowledge level and attitudes of students regarding haemoglobinopathies and their status of being previously informed about the disorders was used.

The sample size was distributed to sub provinces based on population density. Schools participating in the study were determined by simple random method. Schools were listed with their total number of students and school names were thrown into a bag. School names were pulled from the bag until reaching the previously calculated sample size. Following the acquisition of consent from students’ parents, questionnaires were applied by researchers. The schools were re-visited for the 104 absent students (5.4%) the next day and the absent students were also allowed to respond to the questionnaire. Data collection period was seven months between May 2012 and December 2012. At the end of this period, a total of 1925 (96.9%) students filled the questionnaires by face to face method.

### Statistical analysis

Data management and computations of descriptive statistics of the survey were performed using SPSS for Windows software (SPSS, Chicago, IL). Pearson chi-square test and binary logistic regression were applied to assess the results. The level of statistical significance was accepted as p<0.05.

### Ethical considerations

The Ethics Board of Mustafa Kemal University Faculty of Medicine approved this study. On behalf of the children enrolled in our study, written informed consent was obtained from the next of kin.

## Results

A total of 1925 students participated in the study. The educational status of the students’ parents was investigated. 66.9% (1288) of the students’ both parents were at primary school level or lower. For the rest of the students, at least one of the parents had an education level of high school or above.

Evaluating the economic status perception of the students; 7.8% (150) of the students characterized their economic status as very bad or bad, 55.2% (1062) of the students characterized as moderate and 37.0% (713) of the students characterized as good or very good.

15.0% (289) of the students remarked that there was a patient with haemoglobinopathy around them, 79.8% (1536) of the students stated there was no existence of a such patient and 5.2% (100) of the students did not know whether there was a patient with haemoglobinopathy around them.

Of the students who took part, 67.5% (1299) were previously informed about haemoglobinopathies, while 32.5% (626) were not.

**Fig 1:**
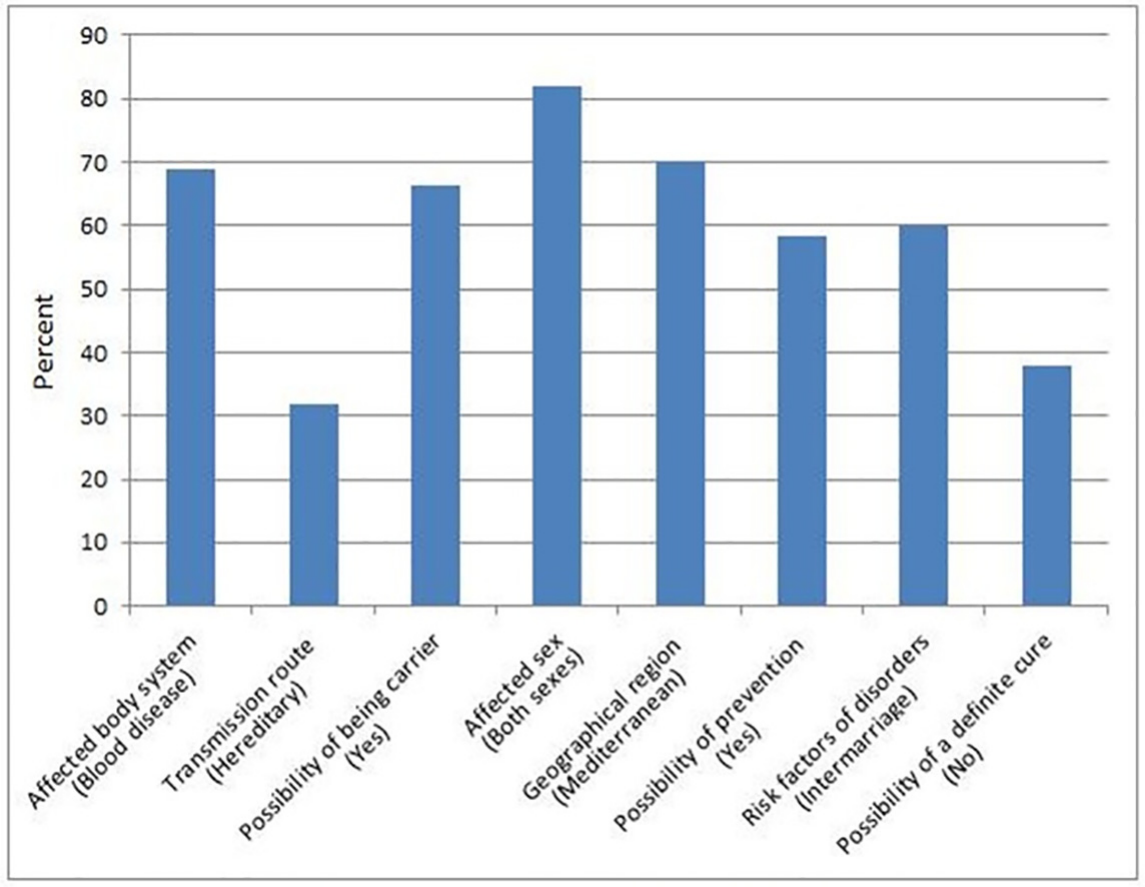
Student’s correct answer rates to the questions regarding haemoglobinopathies.

Among questions regarding students’ knowledge about haemoglobinopathies, the lowest correct response rate was observed in “How do these diseases transmit?” with 31.8% (612), meaning most of the students did not know that the diseases are transmitted by heredity. The most correct response rate was observed in “Which gender do these diseases affect?” with 82.1% (1580) of the students stating the diseases affect both sexes. Students’ correct answer rates to the questions are presented in Fig 1 in detail.

The results revealed that there were significant differences between the correct answer rates of the students and their status of being previously informed, indicating that informing students about the disorders plays an important role in students’ knowledge level about haemoglobinopathies. The correct answer rates of the students according to their status of being previously informed are presented in Table 1.

**Table 1:** Students’ correct answer rate according to their status of being previously informed.

Questions	Correct answer of not previously informed (N = 626)		Correct answer of previously informed (N = 1299)		P-value**
	n	%*	N	%*	
Which system of body is affected from these diseases?	318	50.8	1009	77.7	**0.0001**
How do these diseases transmit?	98	15.7	514	39.6	**0.0001**
Is it possible to be a carrier of these diseases?	315	50.3	961	74.0	**0.0001**
Which gender do these diseases affect?	511	81.6	1069	82.3	0.751
Where in Turkey do these diseases occur most commonly?	361	57.7	991	76.3	**0.0001**
Do you think these diseases are preventable?	347	55.4	776	59.7	0.076
What is an important risk factor for these diseases?	259	41.4	893	68.7	**0.0001**
Do you think that these diseases are curable?	219	35.0	509	39.2	0.079

*The values are in N (%)

**p values are calculated using Pearson chi-square test

87.0% (1675) of the students stated that they wanted to be educated about haemoglobinopathies at school and 81.6% (1570) wanted to learn their carrier status. 70.4% (1356) of the students stated that they would change their future decisions in case these diseases might adversely affect themselves or their family or children in a physical, psychological or social manner. Significant differences were observed between previously informed and not previously informed students in terms of attitude and behavioral pattern. Attitude and behavioral pattern of the students about haemoglobinopathies according to their status of being previously informed is presented in Table 2.

**Table 2:** Attitude and behavioral pattern of students about haemoglobinopathies according to their status of being previously informed.

Attitudes and Behaviors	Not previously informed (N = 626)		Previously informed (N = 1299)		P-value**
	n	%*	n	%*	
Willing to be informed at School	513	81.9	1162	89.5	**0.0001**
Willing to learn their carrier status	488	78.0	1082	83.3	**0.005**
Changes the future plans in case of a risky situation	389	62.1	967	74.4	**0.0001**

*The values are in N (%)

**p values are calculated using Pearson chi-square test

Since the results demonstrated that informing the students about haemoglobinopathies is a key point for increasing knowledge level and changing behavioral pattern of students, a final binary logistic regression analysis was performed to assess the factors affecting the students’ being previously informed status. The variables that were independently and significantly associated with a greater probability of being previously informed were: presence of a patient around, with students who had a diseased person around having a 2.597-fold higher probability of being previously informed (95%CI = 1.886–3.575); economic status perception, with students being in a good or very good economic status having a 1.238-fold higher probability of being previously informed (95%CI = 1.004–1.526); parental education level, with students possessing at least one parent at secondary education level or above having a 1.954-fold higher probability of being previously informed (95%CI = 1.564–2.443). Factors affecting the status of being previously informed are presented in Table 3.

**Table 3:** Factors affecting the status of being previously informed.

Factors				95% C.I.for EXP(B)	
	B	Sig.	Exp(B)	Lower	Upper
**Presence of a patient around**	0.954	**0.000**	2.597	1.886	3.575
**Economic status perception**	0.213	**0.046**	1.238	1.004	1.526
**Parental education level**	0.670	**0.000**	1.954	1.564	2.443
**Constant**	0.329	0.000	1.390		

## Discussion and Conclusion

The struggle against haemoglobinopathies in Turkey officially began by “the law of fight against hereditary blood diseases (no.3960)” published in the Turkish Official Gazette in December 30, 1993 (no.21804) [9]. No sooner was “Congenital Blood Diseases Research and Treatment Centers” founded by Turkish Ministry of Health in the provinces of Hatay, Mersin, Muğla and Antalya, than the screening test for thalassemia was announced obligatory for couples intending to marry. In addition, the “National Haemoglobinopathy Council” was founded with the aims of screening, recording, instruction, antenatal detection and conventional screening [10]. Later, in 2005, the Thalassemia Federation has been founded from the previous National Haemoglobinopathy Council, with the same objectives. The federation adopted the strategy to inform the public on a large scale about haemoglobinopathies using the press, media and associations for public education [6].

The value of the screening programs in reducing the risk of haemoglobinopathy incidence has been firmly established, yet the lack of knowledge and awareness about the disorders and their consequences, may serve as barriers to prevention, disclosure of disease status and testing for haemoglobinopathies [2,3,7].

Eventually, before starting to discuss the results, we would also like to state that, Hatay, being as the study location, is noteworthy from three aspects. Firstly, Hatay is in Cukurova region where haemoglobinopathy prevalence is as high as 10% [4] Secondly, considering Ministry of Health data, the prevalence of abnormal hemoglobin carriers in Hatay is even more, being 15.1% [8]. Thirdly, intermarriage rate in Hatay is up to 34.4% which is quite higher than the Turkey average [11].

In this study, evaluating the answers to the questions that measure the knowledge level of students; the correct answer rates of the students were ranging between 15.7% (about how the disorders are transmitted) and 81.6% (about affected sexes) in those who were not previously informed about the disorders. These rates were higher in those who were previously informed about the disorders being between 39.6% (about how the disorders are transmitted) and 82.3% (about affected sexes), revealing that informing the students about the disorders contributed to a significant increment in the level of knowledge. In a study on university students in Kocaeli, 2007, a significant increase in knowledge about the haemoglobinopathies was observed after an informative lecture [12]. Likewise, it was prerecorded that the participants gained understanding of inheritance and carrying of haemoglobinopathies from an infotainment session in a study performed in high-risk ethnic groups in the Netherlands, in 2009 [13].

The results indicate that about only 30% of the students (not previously informed 15.7%—previously informed 39.6%) from Hatay where haemoglobinopathies are prevalent, could specify that these disorders are transmitted by heredity. In a study conducted on high school students in Antakya, a sub province of Hatay, in 2009, 18.5% of students not tutored on haemoglobinopathies and 44.8% of students tutored on haemoglobinopathies knew that the disorders are hereditary, which is consistent with our findings [6]. Similarly, in a study performed in high schools of Antalya in 1998–1999, it was reported that only 10.7% of the 11^th^ grade students were aware of the inheritance of thalassemia and the results of a marriage of two carriers [14]. In another study performed in 8^th^ class students in Burdur in 2013, 84.3% of the students were aware that thalassemia is not a contagious disease [15]. This rate may seem higher than our study and the studies mentioned above, however in Burdur study, the students were only asked whether thalassemia is contagious or not, instead of making students choose a transmission route from a multiple choice question. In Turkey, studies regarding haemoglobinopathy awareness other than school settings mostly targeted premarital couples. In a study targeting premarital couples executed in Denizli, in 2014, it is reported that 44% of the participants knew that thalassemia passes down through families [16]. In another study carried out in 2015, in Kahramanmaraş, a city not far from Hatay, 46.3% of participants who applied premarital screening were aware that thalassemia is transmitted by heredity [17]. The reason behind premarital couples being more knowledgeable about the hereditary characteristics of haemoglobinopathies was thought to be due to their advanced age and educational status.

Another striking result found in our study is that 58.6% of the students not previously informed on haemoglobinopathies did not know that consanguineous marriage is a risk factor. In accordance with our study, in studies performed on premarital couples, the majority of the participants failed to specify intermarriage as a risk factor [16, 17]. This is particularly important since intermarriage which is frequent in our country, is a contributing factor to high incidence of genetic disorders such as thalassemia and sickle-cell disorder [18–20]. Tunçbilek et al. reported that the rate of consanguinity had been approximately 20–25% for the last 25 years in Turkey and emphasized the necessity of educational interventions in populations which intermarriages are constant [21].

Hopefully, the positive attitude rates of students towards haemoglobinopathies were at satisfactory levels. According to our results; 87.0% of the students stated that they wanted to be educated about haemoglobinopathies at school and 81.6% wanted to learn their carrier status. 70.4% of the students stated that they would change their future decisions in case these diseases might adversely affect themselves or their family or children in a physical, psychological or social manner. Students previously informed about the disorders exhibited a more positive attitude and behavioral pattern towards haemoglobinopathies. Similar results were observed in the study carried out on high school students in Antakya, a sub province of Hatay, in 2009 [6]. In accordance with our study, Miri-Moghaddam et al. reported that positive attitudes of participants were augmented with the increasing knowledge levels in their study conducted on high school students in Iran, in 2009 [22]. Similarly Karimzaei et al. noted that it is essential to raise awareness among the people in order to develop preventive behaviors [3].

The results demonstrated that informing the students about haemoglobinopathies is key point for increasing knowledge level and changing behavioral pattern of students. It is found that higher parental education levels, existence of a patient with haemoglobinopathy and better economic conditions were factors which positively affect the status of being previously informed. Likewise, Miri-Moghaddam et al. reported that with the increment in parents’ educational level, the students’ positive attitude level towards haemoglobinopathies became increased [22] and Savaş et al. reported that the rate of students’ being previously informed status were significantly higher in those who had a diseased person around [6]. Major sources of information about these disorders include health professionals, internet, newspapers, mass media, friends and family members, many of which require certain economic well-being to access [23]. Thus it is not surprising that good economic situation affects the status of being previously informed.

In conclusion, this study pointed out the lack of knowledge about the hereditary characteristics of haemoglobinopathies in middle and high school students and secondly highlighted the importance of raising awareness in terms of preventive behaviors. To render the fight against haemoglobinopathies effective, health education programs including informative lectures about the genetic basis of haemoglobinopathies, genetic counseling and premarital screening should be implemented in the routine curriculum especially in the regions where the disorders are prevalent.

### Strengths and limitations

A strength of the present study is that it was conducted in a city where the disorders are prevalent. Additionally all sub provinces in Hatay were included in the study. Thus, it is quite capable of representing the study universe.

Several limitations should be addressed however. First, due to insufficiency of time and resources, the study was only carried out on the selected sample. Second, as it is a survey study, memory factors that affect the responses to the questionnaire may exist.

## Supporting Information

S1 File Dataset. (SAV)
